# Superior Strength and Ductility of 304 Austenitic Stainless Steel with Gradient Dislocations

**DOI:** 10.3390/nano11102613

**Published:** 2021-10-04

**Authors:** Qingsong Pan, Song Guo, Fang Cui, Lijun Jing, Lei Lu

**Affiliations:** 1 Shenyang National Laboratory for Materials Science, Institute of Metal Research, Chinese Academy of Sciences, Shenyang 110016, China; qspan@imr.ac.cn (Q.P.); sguo19s@imr.ac.cn (S.G.); fcui15s@alum.imr.ac.cn (F.C.); ljjing14s@imr.ac.cn (L.J.); 2 School of Materials Science and Engineering, University of Science and Technology of China, Shenyang 110016, China

**Keywords:** gradient dislocations, mechanical property, low-angle boundary, deformation twinning, stainless steel

## Abstract

Materials with designed gradient nanograins exhibit unprecedented mechanical properties, such as superior strength and ductility. In this study, a heterostructured 304 stainless steel with solely gradient dislocation structure (GDS) in micron-sized grains produced by cyclic-torsion processing was demonstrated to exhibit a substantially improved yield strength with slightly reduced uniform elongation, compared with its coarse grained counterparts. Microstructural observations reveal that multiple deformation mechanisms, associated with the formation of dense dislocation patterns, deformation twins and martensitic phase, are activated upon straining and contribute to the delocalized plastic deformation and the superior mechanical performance of the GDS 304 stainless steel.

## 1. Introduction

Both strong and ductile metallic materials are always desirable, especially crucial for developing high-performance structural components with enhancing service safety and energy efficiency under the current challenge of energy crisis [1,2,3]. Unfortunately, strength and ductility are mutually exclusive [4,5]. Traditional strengthening methodologies are generally based on the generation of various volume or planar defects, such as conventional high-angle grain boundaries (HAGBs, with misorientation angles larger than 15°), and coherent twin boundaries (TBs) to resist the motion and transmission of the intra-grain dislocations [3,4]. In particular, homogeneously reducing grain sizes into the ultrafine or nanometer regime i.e., ultrafine grain or nanograin, (UFG and NG) can substantially elevate the strength and hardness [4]. However, this comes at a dramatically lost tensile ductility, close to zero for most cases [4,6,7]. The tensile brittleness of UFG and NG metals is mainly ascribed to the fact that traditional dislocation-based strain-hardening mechanism becomes invalid, i.e., dislocation slip and accumulation/storage are substantially suppressed in the extremely tiny grains. This, together with severe strain localization with GB migration, results in the absence of work hardening and early necking occurring immediately after yielding [4,7].

Recently, the long-lasting strength-ductility paradox of conventional metallic materials has been circumvented to some extent by engineering spatial heterogeneous nanostructures [8,9,10]. In particular, heterostructured metals with the built-in spatially graded distribution of structural features, such as grain size [8,10], or twin thickness [11,12], spanning from nanometer at surface to microscale at core, exhibit a desirable combination of high strength and considerable ductility, which is not achieved in the non-gradient counterparts [4,7]. Such superior ductility primarily originates from the progressive plastic yielding from the core to surface of the gradient nanostructures, which induces the activation of novel deformation mechanisms [1]. For instance, a mechanically driven GB migration process with grain coarsening in either homogeneous or abnormal mode dominates the plastic deformation of the gradient NG Cu under tension and cyclic deformation [8,13,14,15]. Nevertheless, as for conventional nanosized grains with high-density GBs, structural coarsening inevitably results in the mechanical softening under external mechanical stimuli, which is detrimental to the continuous property increment and technological applications [1,8,16].

How to introduce high-density nanoscale substructure without refining grain size to suppress strain-induced structural coarsening and improve tensile properties is a promising, yet challenging, issue. Austenitic stainless steels are widely used in industry and known to have several plastic deformation mechanisms that include dislocations, twinning and martensitic transformation. Therefore, they are good candidates for this type of research. In this study, by using the cyclic-torsion treatment [17,18], we produce a distinctive gradient nano-scaled low-angle dislocation structure (GDS) without changing initial grain size in one most widespread used 304 stainless steel (304 SS). A remarkably improved yield strength with continuous strain-hardening and high ductility combination is achieved in the GDS 304 SS, owing to the suppressed strain localization and resultant hardening in GDS associated with extensive dislocation storage and deformation twinning. 

## 2. Materials and Methods

Commercial AISI 304 SS rods with the composition of Fe-18.29Cr-8.10Ni-0.061C-0.44Si-1.30Mn-0.006S-0.078P (wt %) produced by Taiyuan Iron & Steel corporation (Taiyuan, China). were initially annealed at 773 K for 1 h to obtain austenitic coarse grains without dislocation (hereafter defined as dislocation-free CG) with an average grain size of 40 μm. Dog-bone-shaped bar CG 304 SS specimens with a gauge diameter of 6 mm and a gauge length of 12 mm were processed by means of a novel cyclic-torsion (CT) treatment on an Instron (Boston, MA, USA) 8874 testing machine at ambient temperature. The CT treatment is referred to as a repeatedly imposed gradient plastic deformation process when one end of the bar sample is torqued under a specific torsion angle amplitude with a number of cycles while the other end is kept fixed [18,19]. The CT processing parameters are described as follows: the torsion angle amplitude of 16° was used at a torsion rate of 144° s^−1^. For each specimen, a torsion number of 200 cycles was imposed to introduce a sample-level homogeneous gradient dislocation structure (GDS).

Uniaxial tensile tests of the GDS and dislocation-free CG specimens were performed on an Instron (Boston, MA, USA) 5982 servohydraulic testing machine at a strain rate of 0.05% s^−1^. An Instron (Boston, MA, USA) 2620-601 static axial clip-on extensometer was used to measure the uniform strain upon loading before necking. To obtain each data set, at least three repeated tensile experiments were performed on each sample. In addition, GDS and dislocation-free CG specimens were interrupted at a tensile strain of 40% and were fully unloaded for microstructural characterization. Microhardness tests of GDS and dislocation-free CG samples before and after tension were performed on a Mitutoyo (Tokyo, Japan) MVK-H3 microhardness tester with a load of 20 g and a holding time of 10 s. The microhardness (*H*v) value at each depth of GDS samples was obtained by averaging 10 measurements, while the error bar is the mean +/− standard deviation (SD). 

The cross-sectional microstructures from the topmost surface to the core of GDS before and after tensile deformation were examined via an FEI (Eindhoven, The Netherlands) Nova Nano460 field emission gun scanning electron microscope (SEM). The electron backscatter diffraction (EBSD) analysis of GDS samples was carried out on Zeiss (Jena, Germany) Supra 55 SEM under a voltage of 20 kV and a current of 6.0 nA with a step size of 500 nm. These foils for SEM and EBSD observations were cut parallel to the dog-bone rod axial direction by an electrical spark machine, and subsequently mechanically polished, then followed by electro-polishing under a voltage of 15 V for ~20 s at room temperature. The cross-sectional microstructures from the topmost surface to the core of GDS before and after tensile deformation were further characterized in an FEI (Eindhoven, The Netherlands) Tecnai G2 F20 transmission electron microscope (TEM) under a voltage of 200 kV. Cross-sectional TEM foils with ~100 μm were sliced along the tensile axis with an electrical spark machine and mechanically polished to a final thickness of 30 μm. After being punched, the TEM foils were fixed to 3 mm diameter Cu rings with a hole of diameter 0.5 mm and thinned by twin-jet polishing in an electrolyte of perchloric acid (8%), alcohol (92%) at −20 °C using a voltage of 30 V. The average size of dislocation cells or walls at different depths of GDS was determined from TEM images on at least 700 measurements.

## 3. Results and Discussions

**Figure 1:**
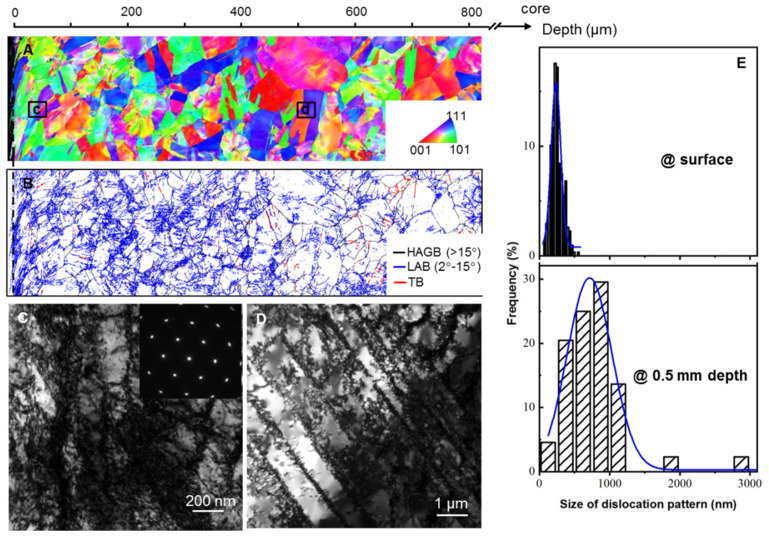
Cross-sectional EBSD images of the gradient dislocation structure (GDS) 304 SS processed by cyclic-torsion processing, showing the distributions of a grain-scaled morphology, orientation (**A**) and LABs (**B**) within an approximately 0.8 mm depth from the surface. The corresponding bright-field TEM images of the deformation structures at the topmost surface (**C**) and ~0.5 mm depth (**D**) of treated samples (indicated in a). The inset in C is the corresponding selected area electron diffraction patterns (SAEDs). (**E**) The corresponding statistic sizes of these dislocation patterns structure at different depths of the GDS 304 SS sample.

Figure 1 shows the cross-sectional microstructure of GDS 304 SS specimens processed by means of CT treatment. Grain orientations from the surface to the core remain random, analogous to that before CT (Figure 1A). No obvious grain refinement with dense HAGBs was detected after CT treatment from the EBSD result. Specially, numerous low-angle boundaries (LABs, with misorientations <15°), as marked by the blue lines in Figure 1B, are introduced in grain interiors, the density of which is spatially decreased with increasing depth from the top surface (Figure 1B). The gradient distributed LABs within unchanged coarse grains after CT treatment are fundamentally distinct from the refined ultrafine or nano grain sizes in traditional homogeneous or gradient nanostructures [4,8,20,21,22,23].

Magnified TEM observations reveal that different dislocation patterns are introduced in grain interiors (Figure 1D), corresponding to massive LABs in Figure 1B. Figure 1C shows numerous dislocation cells at the topmost surface (~0.02 mm) with a high density of dislocations at the cell walls and relatively fewer dislocations in the cell interior. By contrast, with increasing depth to the subsurface (~0.5 mm depth from the topmost surface), the dislocation patterns gradually change to the planar single-slip induced dislocation walls, as shown in Figure 1d, which is universally detected in 304 SS or other faced cubic centered metals with the low stacking fault energy deformed at large strains [3,24]. In contrast, these well-developed dislocation patterns are rarely detected at the core. The local misorientation across dislocation patterns with the LABs is generally accommodated through geometrically necessary dislocations (GNDs) [25,26,27]. With the measured misorientation angle obtained from Figure 1B and the strain-gradient theory [28,29], the GND density per area was estimated to be ~3.1 × 10^14^ m^−2^ in the topmost GDS layer and gradiently decreases with increasing the depth.

Figure 1E shows that the size of dislocation patterns progressively increases, i.e., from 250 nm in the topmost surface to ~850 nm for the dislocation-wall structure at ~0.5 mm depth. Besides, no visible deformation twins and martensitic phase are observed in the GDS sample. Such gradient dislocation structure in the austenitic GDS 304 SS sample is primarily a result of the imposed gradient plastic strain from the topmost surface to the core under a gradient stress/strain state with a large accumulative strain [3,18]. The above microstructural observations indicate that a distinctive hierarchical dislocation structure, i.e., gradiently distributed dislocation patterns and size, is developed in the micron-sized grains of austenitic 304 SS after CT treatment. 

**Figure 2:**
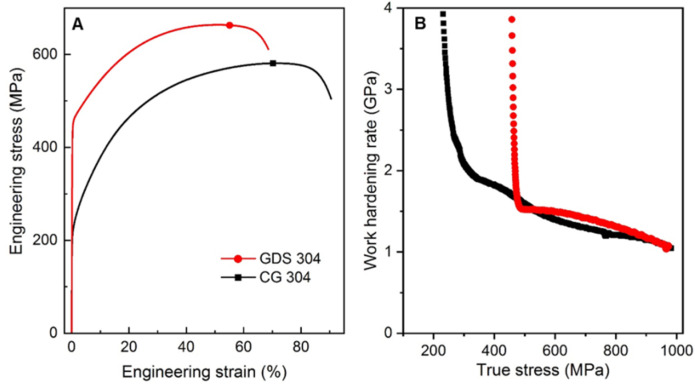
Tensile engineering stress–strain curves (**A**) and the corresponding work hardening rate- true stress relations (**B**) of GDS and dislocation-free CG 304 SS samples.

The typical tensile stress–strain curve of the GDS 304 SS sample in Figure 2 reveals the superior tensile properties.

Its yield strength (*σ*_y_, at 0.2% offset) is measured to be 464 ± 5 MPa, about 2.2 times higher than that of the dislocation-free CG counterpart (210 ± 5 MPa). A uniform elongation (*δ*_u_) up to 55 ± 0.2% is detected, slightly reduced compared to that of the dislocation-free CG counterpart (71 ± 0.9%). Specially, an unexpected strain hardening ability, even slightly higher than that of the dislocation-free CG counterpart at the same stress level, is detected for the GDS sample, which is distinct from the notably reduced Θ trend upon straining in its counterpart with homogeneous dislocation patterns prepared via plastic deformation strategies [3,21,22]. 

**Figure 3:**
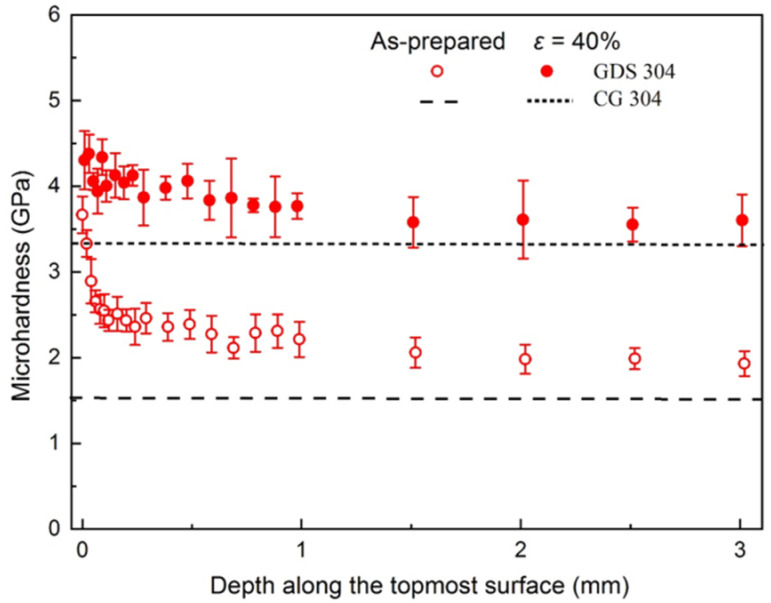
The variations of measured microhardness along the distance from the top surface to the interior of GDS samples after cyclic-torsion processing and those after a tensile strain of 40%. The error bars represent standard deviations from 10 independent hardness measurements. The hardness data of dislocation-free CG 304 SS is also included for comparison.

The microhardness (*H*v) distributions along the depth from the surface to the core of GDS sample before and after tension were measured, as shown in Figure 3. 

Owing to the presence of the sample-level gradient dislocation architecture, the as-prepared GDS 304 SS displays a gradient distributed *H*v, from 3.6 GPa at the topmost surface to 1.9 GPa to the core, much higher than that of dislocation-free CG (~1.5 GPa). Most impressively, tensile strain further leads to monotonically hardening in bulk GDS sample from the topmost surface to the core, which is in contrast to the softening feature in conventional gradient nanograined metals upon straining [8,13,30]. The microhardness in the topmost surface layer and the core of GDS-H is 4.3 and 3.6 GPa after a strain of 40%, exhibiting a *H*v increment of 0.6 and 1.6 GPa, compared to the as-prepared one. 

**Figure 4:**
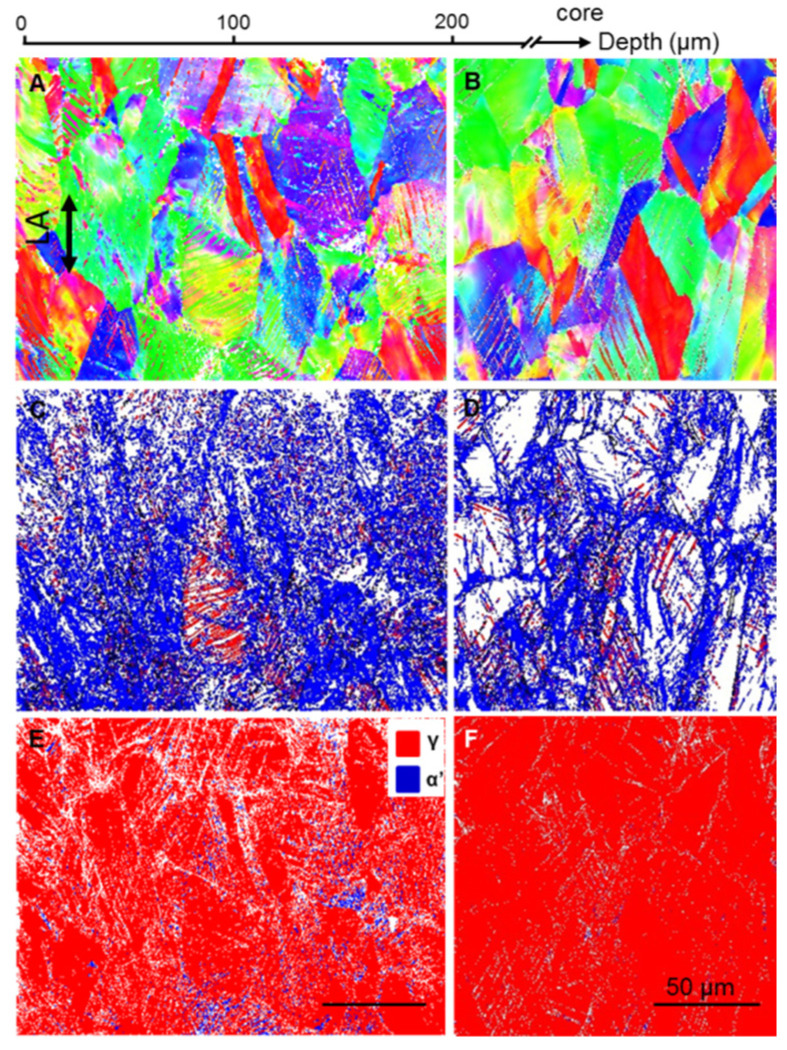
Cross-sectional EBSD images of deformation features in the GDS 304 SS samples at a tensile strain of 40%, showing the distributions of grain-scaled morphology (**A**), LABs (**A**) and phase (**E**) within an approximately 0.2 mm depth from the surface, compared with that in the core (**B**,**D**,**F**).

To explore the underlying strengthening and hardening mechanism of the gradient dislocation structure, we examined the microstructural evolution of GDS 304 SS at the tensile strain of 40% via EBSD, as typically shown in Figure 4.

After tensile deformation, the original coarse grain morphologies at the topmost GDS and core are still kept (Figure 4A,B). The detailed boundary maps further show that the LABs (marked by blue lines in Figure 4C) at the top surface layer become denser and more homogeneous after tension, relative to the homogeneous plastic deformation at the core (Figure 4D). The GND density at the LABs in the topmost GDS layer is measured to be ~4.0 × 10^14^ m^−2^, slightly higher than that before tension. Numerous TBs, denoted by the red lines in Figure 4C, are also prevalently present in the topmost GDS layer, while fewer at the core (Figure 4D), which is the same as the CG counterpart after tensile straining.

In addition, α’-martensite phases are also detected in gradient dislocation structure (Figure 4E), approximately 3% in the volume fraction. Such an enhanced martensitic transformation phenomenon in the topmost GDS structure is in good consistency with its X-ray measurements, where (110) and (211) peaks from martensitic phase are notably detected, with an estimated volume fraction of 11.6% of martensitic. By contrast, fewer detectable martensitic phases exist for the core region of GDS 304 SS at a strain of 40% (Figure 4F), similar to that of the free-standing dislocation-free CG counterpart at the same strain.

**Figure 5:**
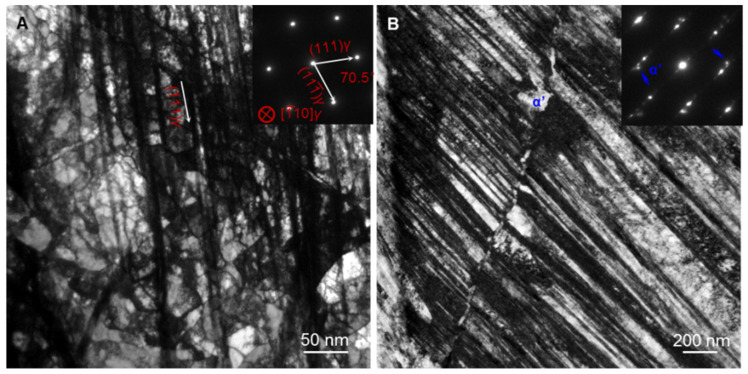
Bright-field TEM images of deformation microstructure of the GDS 304 SS at a tensile strain of 40%, showing the refined dislocation structure (**A**) and the formation of deformation nanotwin and α’-martensite phase (**B**).

The microstructure in topmost GDS structure at the strain of 40% was further characterized by TEM, as shown in Figure 5. Most dislocation cell structures (~70% in the volume fraction) in the topmost GDS layer at the strain of 40% are still detected, yet with a smaller scale (~100 nm), in good agreement with more LABs density in Figure 4C. Specially, dense parallel dislocation bands along the {111} slip plane, ultrafine or nanoscale in inter-width and micron-scale in length, are newly formed and cut through numerous dislocation cells, same to the EBSD results in Figure 4A,C. Many micron-scale-long nanotwin bundles, composed of high-density ultrafine and nanoscale twins, as verified by selected area electron diffraction patterns (Figure 5B), are formed to coordinate the plastic deformation.

Furthermore, the isolated block-shaped α’-martensite phases, scaled at the ~200 nm on average, are present at the intersections of different twin bundles, i.e., white regions marked by the while arrow in Figure 5B, similar to those observed in 304 SS after severe plastic deformation [31,32,33]. The above microstructural observations demonstrate that novel deformation mechanisms associated with the enhanced dislocation interaction, deformation twinning and martensitic transformation are activated in GDS 304 SS. 

Dislocation itself is known as the basic linear defect with local varied strain/stress field to increase the slip resistance of surrounding dislocations; its contribution to the strength is proportionally scaled to the dislocation density, according to the Taylor’s relation [3]. As for GDS, the presence of high density of dislocations themselves is beneficial to strengthening the materials. In addition, LABs have been demonstrated to be as effective in resisting dislocation motion as the conventional HAGBs [34]. As a result, in addition to the high-density dislocations, the nanoscaled dislocation patterns in sample-level GDS strongly block the subsequent inter-cell dislocation slip upon tensile straining, thereby leading to the elevated hardness and yield strength (Figure 2 and Figure 3).

Next, one question that should be addressed is why the hardened surface layer with high density of dislocations deforms in compatibility with the soft core. This is mainly ascribed to the activation of new deformation mechanisms to suppress the possible strain localization, the underlying reason of which will be analyzed below in detail. Different from homogeneous dislocation-free CG, the structural gradient in the heterogeneous nanostructure universally induces progressive plastic deformation from soft core to hard surface under uniaxial tension [1,8]. That results in the macro-scale plastic strain gradient and multiple-axial stress state in the gradient nanostructure [1,10,12,35]. Therefore, the plastic deformation incompatibility is generally accommodated through the generation of GNDs, deformation twinning and other novel dislocation activities, interface-related behavior and interactions between GNDs and interfaces, as shown in Figure 4 and Figure 5. 

Previous studies have demonstrated that α’-martensite preferentially nucleates at the junctions of lamellar nanotwins with different orientations or different microstructures because of the local stress concentration induced by elastic–plastic deformation incompatibility therein [33,36,37,38]. As a result of gradient-induced incompatibility, more martensitic phases were formed in the nanotwin structure at the topmost GDS layer than that at the core of GDS 304 SS (Figure 4 and Figure 5). 

Distinct from the grain coarsening in conventional gradient nanograined metals [1], the new deformation mechanisms of deformation twinning and martensitic transformation as well as extensive dislocation interactions not only mediate the plastic strain, but also progressively induce the structural refinement (Figure 4 and Figure 5), which is in accordance with the monotonically hardening in GDS after tension (Figure 3). With further straining, the resultant progressive structure refinement associated with more intensive dislocation–dislocation pattern and twin interactions contribute to strengthening and strain hardening simultaneously. This explains why the work hardening rate of GDS 304 SS with a high density of pre-existing dislocations is slightly larger than that of dislocation-free CG counterpart at the same stress level (Figure 2B). 

The salient feature of the gradient dislocation structure in this study is the presence of high-density dislocations, which act as ample source for dislocation–dislocation interaction and dislocation storage. Under such circumstance, extensive dislocation interactions with gradual increment in the density of LABs and GNDs take place, as revealed in Figure 4c and Figure 5a, to accommodate the plastic strain and deformation incompatibility in gradient dislocation structure [25,27,28,39].

Thus, the co-activation of multiple plastic deformation mechanisms effectively suppresses the strain localization with the enhanced work hardening rate, resultantly contributing to the considerable uniform elongation of GDS 304 SS at high stress level, compared to dislocation-free CG counterpart (Figure 2A). Impressively, the activation of these distinctive strain-delocalized deformation mechanisms in gradient dislocation structure enables the cost-effective 304 SS exhibiting 160 MPa higher yield strength, yet with comparable uniform elongation (~55%), compared to gradient nanograined 316 SS [40,41]. 

## 4. Conclusions

The gradient dislocation structured 304 SS processed by cyclic-torsion treatment exhibits a 2.2 times higher yield strength while maintaining slightly reduced uniform elongation, compared with its dislocation-free CG counterpart. Such superior mechanical properties primarily stem from the structural gradient induced strain delocalization and the co-activations of various strengthened and hardening deformation mechanisms in GDS, including extensive dislocation interactions and the formation of dense dislocation patterns, nanotwins and martensitic phase. The findings in this study provide a new promising strategy to the design and development of high-performance metallic materials, by tailoring gradient dislocation patterns at the nanoscale. 

## 5. Patents

Two Chinese patents (grant numbers. ZL201911044516.5 and ZL201911043937.6) on the cyclic-torsion processing were granted.

## Data Availability

All data generated or analyzed during this study are included in this published article.
